# Recommendations by Cochrane Review Groups for assessment of the risk of bias in studies

**DOI:** 10.1186/1471-2288-8-22

**Published:** 2008-04-21

**Authors:** Andreas Lundh, Peter C Gøtzsche

**Affiliations:** 1 Nordic Cochrane Centre, Rigshospitalet Dept. 3343, Blegdamsvej 9, DK-2100 Copenhagen Ø, Denmark

## Abstract

**Background:**

Assessing the risk of bias in individual studies in a systematic review can be done using individual components or by summarizing the study quality in an overall score.

**Methods:**

We examined the instructions to authors of the 50 Cochrane Review Groups that focus on clinical interventions for recommendations on methodological quality assessment of studies.

**Results:**

Forty-one of the review groups (82%) recommended quality assessment using components and nine using a scale. All groups recommending components recommended to assess concealment of allocation, compared to only two of the groups recommending scales (P < 0.0001). Thirty-five groups (70%) recommended assessment of sequence generation and 21 groups (42%) recommended assessment of intention-to-treat analysis. Only 28 groups (56%) had specific recommendations for using the quality assessment of studies analytically in reviews, with sensitivity analysis, quality as an inclusion threshold and subgroup analysis being the most commonly recommended methods. The scales recommended had problems in the individual items and some of the groups recommending components recommended items not related to bias in their quality assessment.

**Conclusion:**

We found that recommendations by some groups were not based on empirical evidence and many groups had no recommendations on how to use the quality assessment in reviews. We suggest that all Cochrane Review Groups refer to the Cochrane Handbook for Systematic Reviews of Interventions, which is evidence-based, in their instructions to authors and that their own guidelines are kept to a minimum and describe only how methodological topics that are specific to their fields should be handled.

## Background

The strength of systematic reviews of randomized trials and observational studies, as opposed to narrative reviews and expert opinion, is the application of systematic strategies to reduce bias. Since the conclusion may become unreliable if the data are flawed, this involves an assessment of the internal validity of the included studies [1]. The term methodological quality is often used instead of internal validity, but as quality may address issues that are not related to bias, it would be preferable to speak about an assessment of the risk of bias.

There are four main areas of bias in controlled clinical studies: selection bias (differences in baseline characteristics between the groups of prognostic importance), performance bias (unequal provision of care apart from the treatment under evaluation), detection bias (biased outcome assessment) and attrition bias (biased occurrence and handling of deviations from the protocol and loss to follow-up) [2-8].

The outcome of the risk-of-bias assessment can be listed for the different methodological areas separately (component approach) or by summarizing the information in an overall quality score (scale approach). The risk-of-bias assessment can be used in the review with a variety of approaches. For example, as a threshold for inclusion of studies; as a possible explanation for differences in results between subgroups of studies; by performing sensitivity analyses where only some of the studies are included; or by using a risk-of-bias score as a weight in a meta-analysis of the results.

Using a scale can be tempting but is not well supported by empirical research [9-11]. A major problem with scales is that they often incorporate items that are more related to the quality of reporting, ethical issues or statistical issues than to bias [11].

The biggest producer of systematic reviews, the Cochrane Collaboration, advises against the use of scales [12]. After peer review, the reviews are edited by one of the 51 Cochrane Review Groups related to different fields of healthcare. Most review groups have their own set of instructions to authors, based on the Cochrane Handbook for Systematic Reviews of Interventions [12], and these guidelines are published in the Cochrane Library under the description of the Cochrane Collaboration [13].

There are currently more than 3,000 Cochrane reviews and they have been shown to be of higher methodological quality, on average, than other systematic reviews [14,15]. However, a previous study of 809 Cochrane reviews published from 1995 to 2002 reported that 36% of the review authors had used scales [16]. We examined how the different review groups currently recommend assessment and handling of the risk of bias in the studies, with a focus on the use of scales, and suggest possible improvements.

## Methods

We reviewed the guidelines for assessment of methodological quality of the primary studies included in Cochrane reviews. In March 2007, one author (A.L.) extracted the relevant data from the descriptions of the Cochrane Review Groups in the Cochrane Library, supplemented with information from websites when reference was made to such sites, and with contacts to the review groups to clarify any uncertainties. The other author (P.C.G.) checked the extracted data, and any disagreements were resolved by discussion. Of the 51 review groups, we excluded the Methodology Review Group, as these reviews do not address clinical interventions.

A standardised data sheet was used and data were extracted on:

1) The type of methodological quality assessment recommended for individual studies, i.e. a component or a scale approach.

2) Areas of methodological quality and other areas recommended to be assessed.

3) Recommendations for using methodological quality assessments of individual studies in reviews, e.g. for inclusion of studies or for analytic purposes.

4) Recommendations to grade the level of evidence for the review as a whole.

Six review groups were asked for clarifications and all replied. The Upper Gastrointestinal and Pancreatic Diseases Group was unable to address our questions because it was being reorganized.

Groups that did not provide any recommendations, but referred to the Cochrane Handbook, were classified as recommending a component approach, since the Handbook advises that quality scores should not be used (as this approach is not supported by empirical research, can be time-consuming, and is potentially misleading) [12]. These groups were regarded as having addressed the main areas of bias mentioned in the Handbook: generation of allocation sequence; concealment of allocation; blinding of patients, caregivers and outcome assessors; and follow-up. They were also classified as giving no specific advice for using methodological quality assessments of individual studies in reviews, as the Handbook has no specific recommendations on this. Groups that offered no information and no reference to the Handbook were treated similarly, as we regarded referral to the Handbook as implicit in these cases. Groups that recommended both scales and components as optional were classified as recommending scales (there were only two such groups). Groups that recommended checklists of individual items were classified as recommending components, unless an overall score was calculated. Finally, for groups that recommended specific items in their guidelines but also referred to the Handbook, we assessed what they recommended in their guidelines.

We report the number of groups that recommended scales or components, areas of methodological quality assessed, specific recommendations for using the assessments of individual studies in the reviews, and type of analytical approach recommended, e.g. subgroup or sensitivity analyses, or meta-regression. We used Fisher's exact test to compare proportions [17].

## Results

### Scales and components

Forty-one of the 50 review groups (82%) recommended a component approach, 34 of these explicitly, including 16 which also had reservations about scales (Table 1). Twenty-three of these 41 groups had their own checklists, ranging from 4 to 23 items.

**Table 1:** Type of methodological quality assessment recommended to be used by Cochrane Review Groups. Values are numbers (percentages)

**Type**	**Recommended (n = 50)**
**Component**	**41 (82)**

Component explicitly recommended, reservations towards scales	16 (32)
Component explicitly recommended	18 (36)
Component recommended through quotation of Cochrane Handbook	3 (6)
Component assumed recommended as there was no information	4 (8)

**Scale**	**9 (18)**

Scale explicitly recommended	7 (14)
Scale optional	2 (4)

The remaining nine groups (18%) recommended a scale approach (two as optional to a component approach). Five groups explicitly recommended the Jadad scale, one used it in their model review, one recommended it as optional to a component approach, one recommended different options of checklists and scales that included the Jadad scale, and one explicitly recommended an 11-item checklist used as a scale.

Most review groups recommended assessing the generation of the randomization sequence (70%), concealment of allocation (86%), blinding of patients (84%), caregivers (66%) and outcome assessors (96%), and follow-up (94%) (Table 2). However, only two of the nine groups that recommended scales advised authors to consider concealment of allocation, compared with all 41 groups that recommended components (P < 0.0001). In contrast, all nine groups recommending scales recommended assessment of sequence generation compared to 26 out of 41 groups recommending components (P = 0.04). Furthermore, only one of the groups that recommended scales advised to assess whether the trial authors had conducted an intention-to-treat analysis, compared to 20 groups that recommended components (P = 0.06).

**Table 2:** Areas of methodological quality recommended to be assessed in reviews by Cochrane Review Groups. Values are numbers (percentages)

**Area of quality recommended**	**All (n = 50)**	**Components (n = 41)**	**Scales (n = 9)**
Sequence generation	35 (70)	26 (63)	9 (100)
Concealment of allocation	43 (86)	41 (100)	2 (22)
Blinding of patients	42 (84)	33 (80)	9 (100)
Blinding of caregivers	33 (66)	32 (78)	1 (11)
Blinding of outcome assessors	48 (96)	39 (95)	9 (100)
Follow-up	47 (94)	38 (93)	9 (100)
Intention-to-treat analysis	21 (42)	20 (49)	1 (11)

Four groups (one that recommended scales and three that recommended components) used limits for loss to follow-up (ranging from 10% to 30%) to judge whether attrition bias was avoided. Two review groups regarded blinding as insufficient if the treatment could be identified in more than 20% of the patients because of side effects.

One group, the Back Group, that recommended a scale approach had included items that are not necessarily related to risk of bias in their scale, e.g. similarities between groups at baseline, use of co-intervention, compliance and timing of outcome assessment.

### Content of checklists

The items included in the checklists used by some groups addressed methodological quality, external validity (e.g. inclusion criteria, diagnostic criteria, precision of diagnostic tool, clinical usefulness of the outcome assessment tool, and duration of study), harms (e.g. details of side effects) and statistics (e.g. sample size, power calculation, presentation of the results and appropriateness of the analysis). Four of the 23 checklists contained an assessment of comparability at baseline.

Twenty-eight groups (56%) recommended authors to use the methodological quality assessment of the individual studies in the analyses, one group recommended their use only for descriptive purposes, and the remaining 21 groups gave no recommendations (Table 3). Eight of the nine groups (89%) that recommended scales advised an analytical usage of the assessments, compared to only 20 of the 41 groups (49%) that recommended components (P = 0.06). Twenty-four groups (86%) that recommended an analytical approach advised sensitivity analyses. In addition, seven also recommended the authors to use the methodological quality as a threshold for inclusion of trials and seven recommended subgroup analyses comparing high- and low-quality trials. Six groups recommended to use the quality in a cumulative meta-analysis, as a weight in the meta-analysis, or in meta-regression (Table 4).

**Table 3:** Recommendations by Cochrane Review Groups for using quality assessments of individual studies in reviews. Values are numbers (percentages)

**Approach recommended**	**All (n = 50)**	**Components (n = 41)**	**Scales (n = 9)**
Analytical approach	28 (56)	20 (49)	8 (89)
Descriptive approach	1 (2)	1 (2)	0 (0)
No information	21 (42)	20 (49)	1 (11)

**Table 4:** Type of analytical approach recommended to be used in reviews by Cochrane Review Groups. Values are numbers (percentages)

Type of analytical approach*	**All (n = 28)**	**Components (n = 20)**	**Scales (n = 8)**
Sensitivity analysis	24 (86)	17 (85)	7 (88)
Threshold	7 (25)	4 (20)	3 (38)
Subgroup analysis	7 (25)	4 (20)	3 (38)
Cumulative analysis	3 (11)	1 (5)	2 (25)
Weights	2 (7)	1 (5)	1 (13)
Meta-regression	1 (4)	0 (0)	1 (13)

* More than one answer possible

### Grading the level of evidence

Two groups graded the evidence for the review as a whole. The Back Group recommended using five levels of evidence (no, conflicting, limited, moderate and strong evidence) for qualitative reviews, where data were impossible or too heterogeneous to pool, based on study design and overall study quality. The Musculoskeletal Group recommended four levels of evidence for both qualitative and quantitative reviews based on study design, specific areas of methodological quality and sample size (bronze, silver, gold and platinum).

## Discussion

### Scales

We found that 18% of the Cochrane review groups recommended scales for the methodological quality assessment without any reservations. Our study could suggest that scales may now be used less often, but our results are not directly comparable to those of Moja et al. who found that 36% of the review authors of reviews published from 1995 to 2002 had used scales and that the Jadad scale was most frequently used [16]. Authors may decide to use a component approach although the group recommends a scale, or vice versa, and the numbers of reviews produced are not equally distributed among groups. Even so, we believe the guidelines of the groups are important. Cochrane Reviews are undertaken by authors with different levels of methodological training, and guidelines are probably followed more strictly by less experienced authors. This can be problematic if the guidelines are not in accordance with the empirical research on bias.

**Figure 1:**
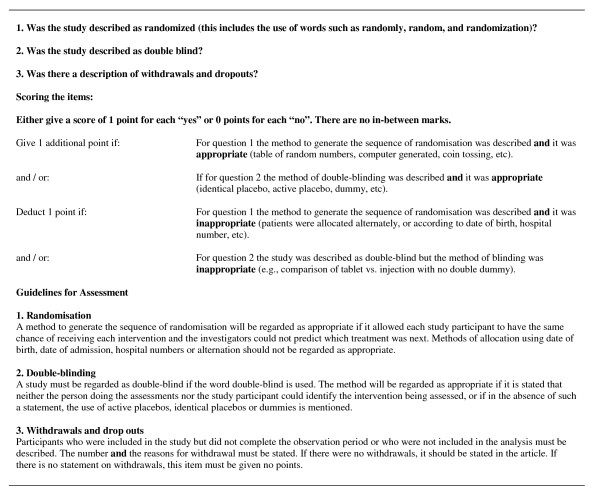
The Jadad scale.

The Jadad scale is the only scale that has been developed using established standards for scales and where low scores have been associated with increased effect estimates [5,9,18,19]. It consists of three items, and up to two points are given for randomization, two for double blinding and one for withdrawals and dropouts (Figure 1). An overall score between zero and five is assigned, where three is commonly regarded as adequate trial quality [18]. Despite its thorough development and validation, the scale is problematic. First, it has more focus on the quality of reporting than on methodological quality [7,8,11]. Second, for randomization, the scale addresses explicitly the sequence generation but not concealment of allocation. The guidelines for the scale state that the investigators should not be able to predict which treatment was next, but this is an implicit way of describing concealment of allocation that may easily be overlooked by the assessors. It is generally considered that the scale does not address this domain [7,8,11]. Third, the scale does not address blinding of caregivers or intention-to-treat analysis. Therefore, randomized trials with no concealment of allocation, no blinding, with large numbers of dropouts that are well described, and with only a per-protocol analysis, may be scored as of good methodological quality (three points). Fourth, studies have shown low interrater agreement, particularly for withdrawals and dropouts, where kappa values below zero have been reported [20,21], which is an agreement that is worse than that expected by chance. Fifth, the Jadad scale – and many other scales – punishes research areas where blinding may be neither feasible, nor relevant, e.g. trials in cancer surgery with total mortality as the main outcome.

Despite advising against scales, the Cochrane Handbook actually recommends a ranking scale [12]. The scale distinguishes between low risk of bias (all criteria met), moderate risk of bias (one or more criteria partly met) and high risk of bias (one or more criteria not met). The types of criteria are not specified, other than they should be few and address substantive threats to the validity of the study results. This scale was recommended explicitly by two groups and implicitly by seven others. In one place, the Handbook states that authors or review groups can use a scale, but that it must be with caution. This is in contrast to the general advice against scales, and this ambiguity can perhaps explain why some groups recommend scales.

The weights and the direction of the bias for the individual items is a substantial problem with scales. As pointed out by Greenland, a true association with two or more components may be overlooked if the associations cancel out in the total score, or if these components have so little weight that this variation is lost in the total score [22]. Usually, all items are given the same weight although it is clear that they do not contribute equally to avoiding bias. For example, the Back Group uses a scale with 11 items, and trials of acceptable quality are defined as those meeting 50% of the criteria (i.e. a minimum of six) [23]. Thus, items on compliance, distribution of co-interventions and timing of outcome assessment are given the same weight as concealment of allocation, which, along with blinding, has been documented as the most important safeguard against bias [3,4]. With this scale, trials that have no concealment of allocation and no blinding can be judged to be of acceptable quality.

The many problems with scales are illustrated in a study by Jüni et al. [11]. These authors used 25 existing scales to identify high-quality trials, and found that the effect estimates and conclusions of the same meta-analysis varied substantially with the scale used.

### Sequence generation

About two-thirds of groups recommended assessing sequence generation. This could be an improvement from the 26% reported previously for Cochrane Reviews [16]. Adequate concealment of allocation may not prevent against selection bias if the sequence generation is deciphered by the persons enrolling patients [4,5,7,24].

### Intention-to-treat analyses

Per-protocol analyses will often lead to substantial overestimation of treatment effects [25-27]. The Cochrane Handbook recommends analyzing all data according to the intention-to-treat principle using different analytical methods such as imputation. Currently it has no recommendations for assessing intention-to-treat analysis as a methodological item or how to assess attrition bias (i.e. loss to follow-up). This is in contrast with 21 groups that recommend to assess intention-to-treat as a separate item using different criteria. While large numbers of loss to follow-up have been associated with bias [6], the use of arbitrarily defined cut-points from 10–30% for assessing attrition bias is not based on empirical results and should therefore not be part of instructions to authors. These findings suggest that the Handbook should give clearer recommendations to ensure a more homogeneous methodology.

### Other problems with scales and items

Several groups recommended assessment of items in their scales or checklists that are hardly related to the risk of bias in clinical studies. For example, the Back Group and the checklists of four other groups recommended to assess for similarities between groups at baseline, but it is not clear how or for what purpose. Proper randomisation ensures that there is no selection bias, but it also means that 5% of baseline characteristics will be expected to differ between the groups at the 5% significance level, and 1% at the 1% level, etc. Furthermore, significant differences in some characteristics may have no effect on the outcome while non-significant differences in others may. Statistical hypothesis testing of the distribution of baseline characteristics should therefore usually only be performed if fraud is suspected [28,29]. It can also be problematic to assess the use of co-interventions and the level of compliance, as both of these may merely reflect the differential effects of the studied interventions.

Another example is the Moncrief scale that is used by the Depression, Anxiety and Neurosis Group as a checklist, without assigning an overall score as was originally the intention [30]. This scale has 23 items and some relate to external validity and appropriateness and reporting of statistical analysis, which are not associated with bias in the study. As chance findings can be misinterpreted as bias, such items can be problematic not only in a scale approach but also in a component approach, if they are used as a threshold for inclusion of studies in the review or in a sensitivity analysis.

The Peripheral Vascular Diseases Group referred to the "Schulz scale", but their reference includes no such scale [4], and Schulz has never constructed one; in fact, he advises against the use of scales for assessment of methodological quality. The Drugs and Alcohol Group recommended against assessing detection bias because of low interobserver agreement, but did not document this statement. The Incontinence Group and the Heart Group described attrition bias as selection bias occurring after randomization, which, although not formally incorrect, is confusing, as it is well understood that selection bias is avoided by proper randomization.

The Musculoskeletal Group recommended a scale for quality assessment of non-randomized studies [31]. The problems with scales are likely much greater for non-randomized studies than for randomized trials, as there is not much empirical evidence for the degree of bias, on average, that is introduced if different criteria are not met.

### Usage of methodological quality assessments

Only a little more than half of the groups had recommendations for using the quality assessment in reviews. The analytical method most often endorsed was sensitivity analysis to test if including only trials of higher methodological quality changes the effect estimates. As explained above, such analyses should not be based on an overall score. Rather than accepting the different combinations of criteria that are possible using scales, one should use one criterion, or only a few important ones simultaneously. For example, in a Cochrane review where a main outcome was number of blood transfusions [32], which is vulnerable to bias if the trial is not blinded, high-quality trials were defined as those that had adequate concealment of allocation and double blinding. Furthermore, high- and low-quality trials were grouped separately in the meta-analyses for easy comparisons.

It is also questionable to exclude trials entirely from the review if they fall below a certain quality cut-point on a scale [24], whereas it can be entirely reasonable to include only trials that are adequately randomized and blinded, e.g. if the main outcome is subjective, such as pain.

### Grading of the evidence for the whole review

Grading of the evidence can help guide the decisions of clinicians and patients [33], provided the grading system is logically consistent and is in accordance with results from empirical studies. The grading system recommended by the Back Group has five levels of evidence and was developed using a consensus method [23]. Consistent findings among multiple, low-quality non-randomized studies are considered to be the same level of evidence as one high-quality randomized trial, which is not in accordance with findings from empirical studies [34,35], or with the Cochrane Handbook [12]. Consistent results from non-randomized studies may merely reflect that they are all biased to a similar degree. This was the case, for example, for hormone replacement therapy, where a meta-analysis of observational studies [36] as well as a large cohort study [37] showed that hormones decreased the incidence of coronary heart disease by about 50%, whereas a high-quality randomized trial showed that hormones cause heart disease [38]. The Back Group intends to remove this scale from its guidelines [39] and will use the GRADE system for grading evidence [40,41].

The four-level grading system used by the Musculoskeletal Group is also based on consensus [42] and is also highly problematic. The system is based on arbitrary cut-points such as sample size above 50 and more than 80% follow-up, which are not based on empirical evidence. The only difference between platinum and gold evidence is that there needs to be two randomized trials for platinum and one for gold, which is not reasonable, as, for example, the platinum trials could involve 60 patients each and the gold trial 500 patients. Silver level can be either a randomized trial with a 'head-to-head' comparison of agents or a high-quality case-control study, which is hard to accept, and bronze level can be a high-quality case series without controls or expert opinion.

## Conclusions

The Cochrane Handbook is produced by experts in methodology, is evidence-based, and is regularly updated in accordance with new evidence. The long guidelines of some review groups therefore seem to be superfluous, and in some cases they are not in accordance with the Handbook, or with the empirical evidence on bias. As the guidelines are probably followed by many review authors, they could potentially threaten the credibility of the reviews. We suggest that all Cochrane Review Groups refer to the Cochrane Handbook in their instructions to authors and that their own guidelines are kept to a minimum and describe only how methodological topics that are specific to their fields should be handled.

The Cochrane Handbook is currently being updated to ensure a more homogenous methodology in its reviews [43]. This revision is based on the acknowledgement of the discrepancies in assessment of methodological quality between the review groups [44], and it will involve introduction of a detailed risk-of-bias tool to be used in all reviews. The tool will also address bias in selective outcome reporting [45,46]. Finally, we suggest that the revision should improve recommendations for assessing attrition bias and the usage of the risk-of-bias assessments, as the current recommendations are not clear about this.

## Competing interests

Both authors work at The Nordic Cochrane Centre and A.L. is currently doing a study in association with the Cochrane Childhood Cancer Group.

## Authors' contributions

Both authors conceived the study and wrote the draft protocol and draft manuscript. A.L. developed data extraction sheets and extracted data, P.C.G. checked extracted data and did the statistical analyses. Both authors contributed to study design, acquisition and interpretation of data and writing the paper. Both authors are guarantors.

## Disclaimer

The views expressed in this article represent those of the authors and are not necessarily the views or the official policy of the Cochrane Collaboration.

## Pre-publication history

The pre-publication history for this paper can be accessed here:



## References

[B1] Egger M, Smith GD, Sterne JA (2001). Uses and abuses of meta-analysis. Clin Med.

[B2] Feinstein AR (1985). An outline from cause-effect evaluations. Clinical Epidemiology: The Architecture of Clinical Research.

[B3] Pildal J, Hróbjartsson A, Jørgensen KJ, Hilden J, Altman DG, Gøtzsche PC (2007). Impact of allocation concealment on conclusions drawn from meta-analyses of randomized trials. Int J Epidemiol.

[B4] Schulz KF, Chalmers I, Hayes RJ, Altman DG (1995). Empirical evidence of bias. Dimensions of methodological quality associated with estimates of treatment effects in controlled trials. JAMA.

[B5] Moher D, Pham B, Jones A, Cook DJ, Jadad AR, Moher M, Tugwell P, Klassen TP (1998). Does quality of reports of randomised trials affect estimates of intervention efficacy reported in meta-analyses?. Lancet.

[B6] Tierney JF, Stewart LA (2005). Investigating patient exclusion bias in meta-analysis. Int J Epidemiol.

[B7] Kjaergard LL, Villumsen J, Gluud C (2001). Reported methodologic quality and discrepancies between large and small randomized trials in meta-analyses. Ann Intern Med.

[B8] Egger M, Jüni P, Bartlett C, Holenstein F, Sterne J (2003). How important are comprehensive literature searches and the assessment of trial quality in systematic reviews? Empirical study. Health Technol Assess.

[B9] Moher D, Cook DJ, Jadad AR, Tugwell P, Moher M, Jones A, Pham B, Klassen TP (1999). Assessing the quality of reports of randomised trials: implications for the conduct of meta-analyses. Health Technol Assess.

[B10] Emerson JD, Burdick E, Hoaglin DC, Mosteller F, Chalmers TC (1990). An empirical study of the possible relation of treatment differences to quality scores in controlled randomized clinical trials. Control Clin Trials.

[B11] Jüni P, Witschi A, Bloch R, Egger M (1999). The hazards of scoring the quality of clinical trials for meta-analysis. JAMA.

[B12] Higgins JPT, Green S, editors (2006). Assessment of study quality. Cochrane Handbook for Systematic Reviews of Interventions 4.2.6 [updated September 2006]; Section 6. The Cochrane Library.

[B13] Cochrane Review Groups (CRGs). http://www.mrw.interscience.wiley.com/cochrane/cochrane_clabout_contents_fs.html.

[B14] Jadad AR, Cook DJ, Jones A, Klassen TP, Tugwell P, Moher M, Moher D (1998). Methodology and reports of systematic reviews and meta-analyses: a comparison of Cochrane reviews with articles published in paper-based journals. JAMA.

[B15] Jørgensen AW, Hilden J, Gøtzsche PC (2006). Cochrane reviews compared with industry supported meta-analyses and other meta-analyses of the same drugs: systematic review. BMJ.

[B16] Moja LP, Telaro E, D'Amico R, Moschetti I, Coe L, Liberati A (2005). Assessment of methodological quality of primary studies by systematic reviews: results of the metaquality cross sectional study. BMJ.

[B17] Langsrud Ø Fisher's exact test. http://www.matforsk.no/ola/fisher.htm.

[B18] Jadad AR, Moore RA, Carroll D, Jenkinson C, Reynolds DJ, Gavaghan DJ, McQuay HJ (1996). Assessing the quality of reports of randomized clinical trials: is blinding necessary?. Control Clin Trials.

[B19] Moher D, Jadad AR, Nichol G, Penman M, Tugwell P, Walsh S (1995). Assessing the quality of randomized controlled trials: an annotated bibliography of scales and checklists. Control Clin Trials.

[B20] Bhandari M, Richards RR, Sprague S, Schemitsch EH (2001). Quality in the reporting of randomized trials in surgery: is the Jadad scale reliable?. Control Clin Trials.

[B21] Clark HD, Wells GA, Huet C, McAlister FA, Salmi LR, Fergusson D, Laupacis A (1999). Assessing the quality of randomized trials: reliability of the Jadad scale. Control Clin Trials.

[B22] Greenland S (1994). Quality scores are useless and potentially misleading. Am J Epidemiol.

[B23] van Tulder M, Furlan A, Bombardier C, Bouter L, Editorial Board of the Cochrane Collaboration Back Review Group (2003). Updated method guidelines for systematic reviews in the cochrane collaboration back review group. Spine.

[B24] Jüni P, Altman DG, Egger M (2001). Systematic reviews in health care: Assessing the quality of controlled clinical trials. BMJ.

[B25] Lewis JA, Machin D (1993). Intention to treat-who should use ITT?. Br J Cancer.

[B26] Melander H, Ahlqvist-Rastad J, Meijer G, Beermann B (2003). Evidence b(i)ased medicine-selective reporting from studies sponsored by pharmaceutical industry: review of studies in new drug applications. BMJ.

[B27] Melander H (2005). [Selective reporting-greater problem than selective publishing?]. Läkartidningen.

[B28] Altman DG (1985). Comparability of randomised groups. Statistician.

[B29] Schulz KF, Chalmers I, Grimes DA, Altman DG (1994). Assessing the quality of randomization from reports of controlled trials published in obstetrics and gynecology journals. JAMA.

[B30] Moncrieff J, Churchill R, Drummond DC, McGuire H (2001). Development of a quality assessment instrument for trials of treatment for depression and neurosis. Int J Methods Psychiatr Res.

[B31] Wells GA, Shea B, O'Connell D, Peterson J, Welch V, Losos M, Tugwell P The Newcastle-Ottawa Scale (NOS) for assessing the quality of nonrandomised studies in meta-analyses. http://www.ohri.ca/programs/clinical_epidemiology/oxford.htm.

[B32] Gøtzsche PC, Hróbjartsson A (2005). Somatostatin analogues for acute bleeding oesophageal varices. Cochrane Database Syst Rev.

[B33] Glasziou P, Vandenbroucke JP, Chalmers I (2004). Assessing the quality of research. BMJ.

[B34] Kunz R, Vist G, Oxman AD (2007). Randomisation to protect against selection bias in healthcare trials. Cochrane Database Syst Rev.

[B35] Deeks JJ (2007). Systematic reviews evaluating effects of health care interventions: Issues of synthesis and bias. [Dissertation].

[B36] Stampfer MJ, Colditz GA (1991). Estrogen replacement therapy and coronary heart disease: a quantitative assessment of the epidemiologic evidence. Prev Med.

[B37] Stampfer MJ, Colditz GA, Willett WC, Manson JE, Rosner B, Speizer FE, Hennekens CH (1991). Postmenopausal estrogen therapy and cardiovascular disease. Ten-year follow-up from the nurses' health study. N Engl J Med.

[B38] Rossouw JE, Anderson GL, Prentice RL, LaCroix AZ, Kooperberg C, Stefanick ML, Jackson RD, Beresford SA, Howard BV, Johnson KC, Kotchen JM, Ockene J (2002). Risks and benefits of estrogen plus progestin in healthy postmenopausal women: principal results From the Women's Health Initiative randomized controlled trial. JAMA.

[B39] Updated Methods Guidelines. Cochrane Back Group June 2007 Newsletter.

[B40] Atkins D, Best D, Briss PA, Eccles M, Falck-Ytter Y, Flottorp S, Guyatt GH, Harbour RT, Haugh MC, Henry D, Hill S, Jaeschke R, Leng G, Liberati A, Magrini N, Mason J, Middleton P, Mrukowicz J, O'Connell D, Oxman AD, Phillips B, Schünemann HJ, Edejer TT, Varonen H, Vist GE, Williams JW, Zaza S (2004). Grading quality of evidence and strength of recommendations. BMJ.

[B41] Grading the quality of evidence and the strength of recommendations. http://www.gradeworkinggroup.org/intro.htm.

[B42] Tugwell P, Shea B, Boers M, Brooks P, Simon L, Strand V, Wells G (2004). Introduction. Evidence-based Rheumatology London: Blackwell BMJ Books.

[B43] Higgins J, Altman D (2007). Assessing quality of included studies in Cochrane reviews. The Cochrane Collaboration Methods Groups Newsletter.

[B44] Higgins J, Hopewell S (2005). Bias susceptibility in Cochrane reviews. Cochrane News.

[B45] Gøtzsche PC (1989). Methodology and overt and hidden bias in reports of 196 double-blind trials of nonsteroidal antiinflammatory drugs in rheumatoid arthritis. Control Clin Trials.

[B46] Chan A-W, Hróbjartsson A, Haahr MT, Gøtzsche PC, Altman DG (2004). Empirical evidence for selective reporting of outcomes in randomized trials: comparison of protocols to published articles. JAMA.

